# Long noncoding RNA DIO3OS interacts with miR-122 to promote proliferation and invasion of pancreatic cancer cells through upregulating ALDOA

**DOI:** 10.1186/s12935-019-0922-y

**Published:** 2019-07-30

**Authors:** Kang Cui, Shuiling Jin, Yabing Du, Junlin Yu, Han Feng, Qingxia Fan, Wang Ma

**Affiliations:** Department of Oncology, The First Affiliated Hospital of Zhengzhou University, No 1. Jianshe Road, Erqi District, Zhengzhou, Henan 450052 China

**Keywords:** DIO3OS, miR-122, ALDOA, Pancreatic cancer

## Abstract

**Background:**

Long noncoding RNA (lncRNA) has been implicated in numerous tumors, including pancreatic cancer (PC). However, the precise cellular roles and molecular mechanisms of lncRNA DIO3OS on PC development remains to be fully clarified.

**Methods:**

We performed the meta-analysis on PC samples and non-tumor samples retrieved from the TCGA database, and measured the levels of DIO3OS in PC cell lines and a normal pancreatic duct epithelial cell line HPDE6-C7. Cell proliferation was evaluated via CCK-8 assay. Cell invasion in vitro was investigated by transwell assay. The RNA immunoprecipitation assay and luciferase reporter assay was utilized to confirm the putative miR-122-binding site in DIO3OS. The effects of DIO3OS on PC progression were tested using in vivo subcutaneous xenografts.

**Results:**

Our results showed that DIO3OS was highly expressed in human PC tissues and PC cell lines. DIO3OS exhibited oncogenic properties in stimulating PC cell proliferation and invasion in vitro and promoting cancer growth in vivo. Through online predictive tools and functional experiments, we found that DIO3OS could bind directly to microRNA-122 (miR-122) and inhibited its expression, which functioned as a tumor suppressor in PC cells. We also verified that ALDOA was the direct target of miR-122, and the tumor suppressive effects caused by DIO3OS knockdown or miR-122 overexpression could be rescued by re-expression of ALDOA in PC cells.

**Conclusions:**

Overall, our study suggested that lncRNA DIO3OS promotes PC cell growth and invasion by competing for miR-122 to modulate the expression of ALDOA. These findings yield a better understanding of the potential mechanisms by which gain of DIO3OS expression accelerates PC progression.

## Background

Pancreatic cancer (PC) is one of the deadliest tumors with a very low 5-year survival rate (ranges from 2 to 9%) [1]. Although surgical resection provides a potential cure, about 70% of patients still develop early recurrence within 6–12 months following surgery [1]. Obviously, the identification of the molecular mechanisms in the initiation and progression of PC is critical for the development of various strategies for PC.

Long non-coding RNAs (lncRNAs) are dysregulated in multiple human cancers including PC [2], and have been implicated in the control of cellular proliferation, apoptosis, differentiation, migration and invasion. LncRNAs can act as signals, decoys, guides, scaffolds or competing endogenous RNAs (ceRNAs) to modulate gene expression [3]. DIO3OS is an antisense lncRNA transcribed from the DIO3 gene imprinted locus [4]. However, the role and the molecular mechanisms of DIO3OS in PC remain to be delineated.

In the present study, we found that lncRNA DIO3OS was significantly upregulated in PC tissues and PC cell lines. Moreover, knockdown of DIO3OS expression suppressed PC cell proliferative and invasion, while overexpression of DIO3OS in PC cells was sufficient to stimulate cell proliferation and invasion. Upon further mechanistic examination, we revealed that DIO3OS served as a molecular sponge for miR-122 to upregulate the expression of ALDOA, which promoted PC cell proliferation and invasion.

## Methods

### Cell culture and transfection

Human PC cell lines (AsPC-1, MIA PaCa-2, PANC-1 and BxPC-1) and human pancreatic duct epithelial cell line HPDE6-C7 were purchased from the American Type Culture Collection (Rockville, USA). The cells were grown in RPMI1640 or DMEM (Gibco, USA) supplemented with 10% fetal bovine serum (FBS). Plasmids containing DIO3OS and ALDOA, or an empty vector pcDNA3.1 were obtained from Genepharma (Shanghai, China). DIO3OS siRNA, control siRNA miR-122 mimic, control mimic, miR-122 inhibitor and control inhibitor were purchased from IGEbio (Guangzhou, China). Lipofectamine 2000 (Invitrogen, Carlsbad, CA, USA) was used for cell transfection following the manufacturer’s instructions.

### Real-time quantitative PCR (qRT-PCR)

Total RNA was isolated from cells using TRIzol reagent (Invitrogen, Carlsbad, CA, USA) and then was converted to cDNA using an M-MLV Reverse Transcriptase Kit (Invitrogen, Carlsbad, CA, USA). Real-time PCR analysis was carried out using the SYBR-Green-quantitative real-time PCR Master Mix kit (Toyobo, Osaka, Japan). The primers for DIO3OS and GAPDH have been reported [5]. GAPDH served as the endogenous control. For detecting miRNA expression, the mirVanaTM qRT-PCR microRNA Detection Kit (Ambion Inc., Austin, TX, USA) was used according to the manufacturer’s instructions. MiR-122 expression was normalized to U6.

### Western blot analysis

Total protein from cells was extracted using RIPA buffer (Beyotime). An equal amount of each protein sample was separated on a 10% SDS-PAGE gel and transferred to a PVDF membrane (Millipore, Bedford, MA, USA). The membranes were blocked with 5% nonfat milk at room temperature for 1 h and incubated with specific primary antibody, ALDOA (1:2000, Abcam, Cambridge, UK) and GAPDH (1:5000, Santa Cruz, CA, USA) overnight, followed by incubation with HRP-conjugated secondary antibodies (Santa Cruz, CA, USA). The protein bands were detected using ECL western blotting kit (Amersham Biosciences, Buckinghamshire, UK). GAPDH was used as the loading control.

### Cell proliferation assay

Cell proliferation was measured by performing the CCK-8 assay (Beyotime Institute of Biotechnology, Jiangsu, China) according to the manufacturer’s instructions. 5000 cells were seeded into a 96-well plate and were transfected as indicated. Then, cell proliferation was measured 72 h after transfection. The absorbance was measured at 450 nm by a microplate reader (Bio-Rad, Hercules, CA, USA).

### Colony formation assay

A total of 1000 cells were seeded in 24-well plates. After culturing for 14 days, colonies were fixed with 100% methanol for 15 min and then stained with 0.5% crystal violet for 20 min. Colonies with > 100 cells were counted and analyzed.

### Cell invasion assay

Transwell invasion assay was performed according to the method described previously [6, 7]. Briefly, 2 × 10^4^ cells in serum-free medium were plated into the upper chamber. The medium containing 10% FBS was added to the lower chamber. After culturing for 24 h, Cell that had invaded were fixed with 75% methanol and stained with crystal violet. Evaluation of invasive capacity was performed by counting invaded cells under a microscope, and five random fields of view were analyzed for each chamber.

### Luciferase reporter assay

A luciferase reporter assay was performed as previously described [8]. The fragment from wild-type DIO3OS (DIO3OS-WT) containing the predicted miR-122-binding site, mutant DIO3OS (DIO3OS-MUT), the 3′-UTR fragment from wild-type ALDOA 3′-UTR (ALDOA-WT) containing the potential miR-122-binding site and mutant ALDOA 3′-UTR (ALDOA-MUT) were amplified using PCR and sub-cloned into a pMIR-GLO Luciferase vector (Promega, Madison, WI, USA). PC cells were co-transfected with the above luciferase reporter vectors containing DIO3OS (WT or MUT) or ALDOA 3′-UTR (WT or MUT) together with miR-122 mimic, miR-122 inhibitor or their negative controls using Lipofectamine 2000 (Invitrogen). The relative luciferase activity was measured with the Dual-Luciferase Reporter Assay System (Promega, China) after 48 h.

### RNA immunoprecipitation (RIP) assay

RNA immunoprecipitation assay was conducted using the Magna RIP RNA-Binding Protein Immunoprecipitation Kit (Millipore) as previously described [9, 10]. Briefly, anti-Argonaute2 (Ago2) antibody (Millipore, Bedford, MA, USA) or normal mouse IgG (Millipore) as a negative control were conjugated to magnetic beads and were incubated with the cell extract in RIP buffer. The immunoprecipitated RNAs were isolated and were subjected to qRT-PCR analysis of DIO3OS and miR-122 expression.

### Xenograft assay

All animal procedures were approved by the Institutional Animal Care and Use Committee of First Affiliated Hospital of Zhengzhou University. Female BALB/c nude mice (4 weeks old) were purchased from Beijing HFK Bioscience (Beijing, China) and maintained under pathogen-free conditions.

To evaluate the in vivo tumorigenic effects, MIA PaCa-2 and AsPC-1 cells (2 × 10^6^) were inoculated subcutaneously in the right flank of the nude mice (n = 4 per group). After implantation for 6 days, tumor volume measurement began and was performed every 3 days, using the following formula: volume = length (mm) × width^2^ (mm^2^)/2. After 3 weeks, the mice were sacrificed and the tumors were collected for immunohistochemistry assay. The xenografts tissues were formalin-fixed/paraffin-embedded and cut into 4 μm slides. The primary antibody used was anti-Ki-67 (1:1000, Abcam, Cambridge, UK). The secondary streptavidin–horseradish peroxidase-conjugated antibody staining was performed at room temperature, visualized in 3, 3′-diaminobenzidine (ZLI9018, ZSGBBIO, China).

### Statistical analysis

Statistical analyses were performed using SPSS 17.0 statistical software (SPSS, Chicago, USA). All data are expressed as the mean ± standard deviation. The Student’s t-test or one-way ANOVA test was used to analyze the significant differences. All experiments were done at least three times. *P*-value < 0.05 was considered to be statistically significant.

## Results

### Levels of DIO3OS are frequently higher in PC tissues and cell lines

**Fig. 1:**
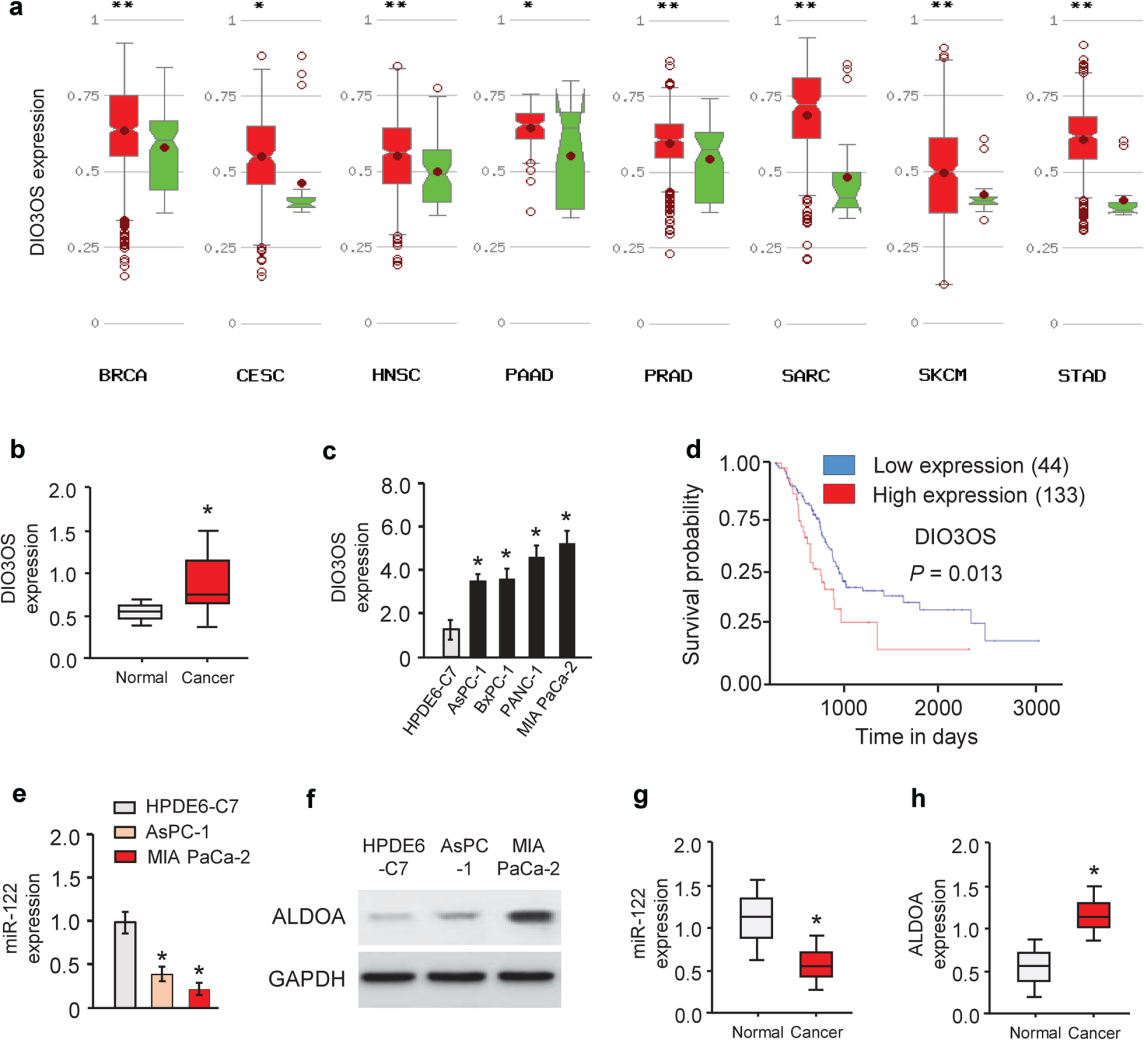
DIO3OS expression is up-regulated in human PC tissues and PC cell lines. **a** DIO3OS expression pattern was analyzed in a panel of cancer (red) vs. normal (green) tissues from the MethHC database. Pancreatic adenocarcinoma: PAAD. **b** Expression of DIO3OS in PC samples and normal pancreatic tissues. The Cancer Genome Atlas (TCGA) datasets were retrieved in the UALCAN web server. **c** qRT-PCR analysis of DIO3OS levels in four PC cell lines and normal pancreatic cell line HPDE6-C7. **d** Kaplan–Meier curves for overall survival of PC patients were compared between groups with high or low levels of DIO3OS. **e** Expression of miR-122 in two PC cell lines and normal pancreatic cell line HPDE6-C7 was examined using qRT-PCR analysis. **f** Western blot analysis of ALDOA expression in PC cell lines and normal cell line HPDE6-C7. The membranes were cut prior to exposure so that only the portion of gel containing desired bands would be visualized. **g** Expression of miR-122 in PC samples and normal pancreatic tissues. The Cancer Genome Atlas (TCGA) datasets were retrieved from the MethHC database. **h** The levels of ALDOA in PC samples and normal pancreatic tissues. The Cancer Genome Atlas (TCGA) datasets were retrieved using the UALCAN web server. *P < 0.05. **P < 0.005

First, we analyzed the TCGA expression data of DIO3OS using the MethHC database (http://methhc.mbc.nctu.edu.tw/php/index.php). DIO3OS was highly overexpressed in a variety of cancer types (including PC) as compared to the corresponding normal tissues (Fig. 1a). Then, we performed the meta-analysis on PC samples and non-tumor samples retrieved from the TCGA database using the UALCAN web server [11]. We found that the DIO3OS transcript was remarkably increased in PC tissues compared with normal samples (Fig. 1b). Furthermore, we measured the levels of DIO3OS in four PC cell lines (AsPC-1, MIA PaCa-2, PANC-1 and BxPC-1 cells) and a normal pancreatic duct epithelial cell line HPDE6-C7. Our qRT-PCR experiments suggested that DIO3OS expression was significantly elevated in PC cells compared to HPDE6-C7 cells (Fig. 1c). Furthermore, we surveyed the association between DIO3OS levels and overall survival in TCGA PC dataset using the web portal of UALCAN (http://ualcan.path.uab.edu/index.html). A total of 177 PC patients with RNA sequencing data and overall outcome data were used for Kaplan–Meier survival analysis. The patients with higher level of DIO3OS displayed shorter overall survival period (Fig. 1d). These data implied that increased expression of DIO3OS may be associated with tumorigenesis or progression of PC.

### Overexpression of DIO3OS promotes PC cell proliferation and invasion

**Fig. 2:**
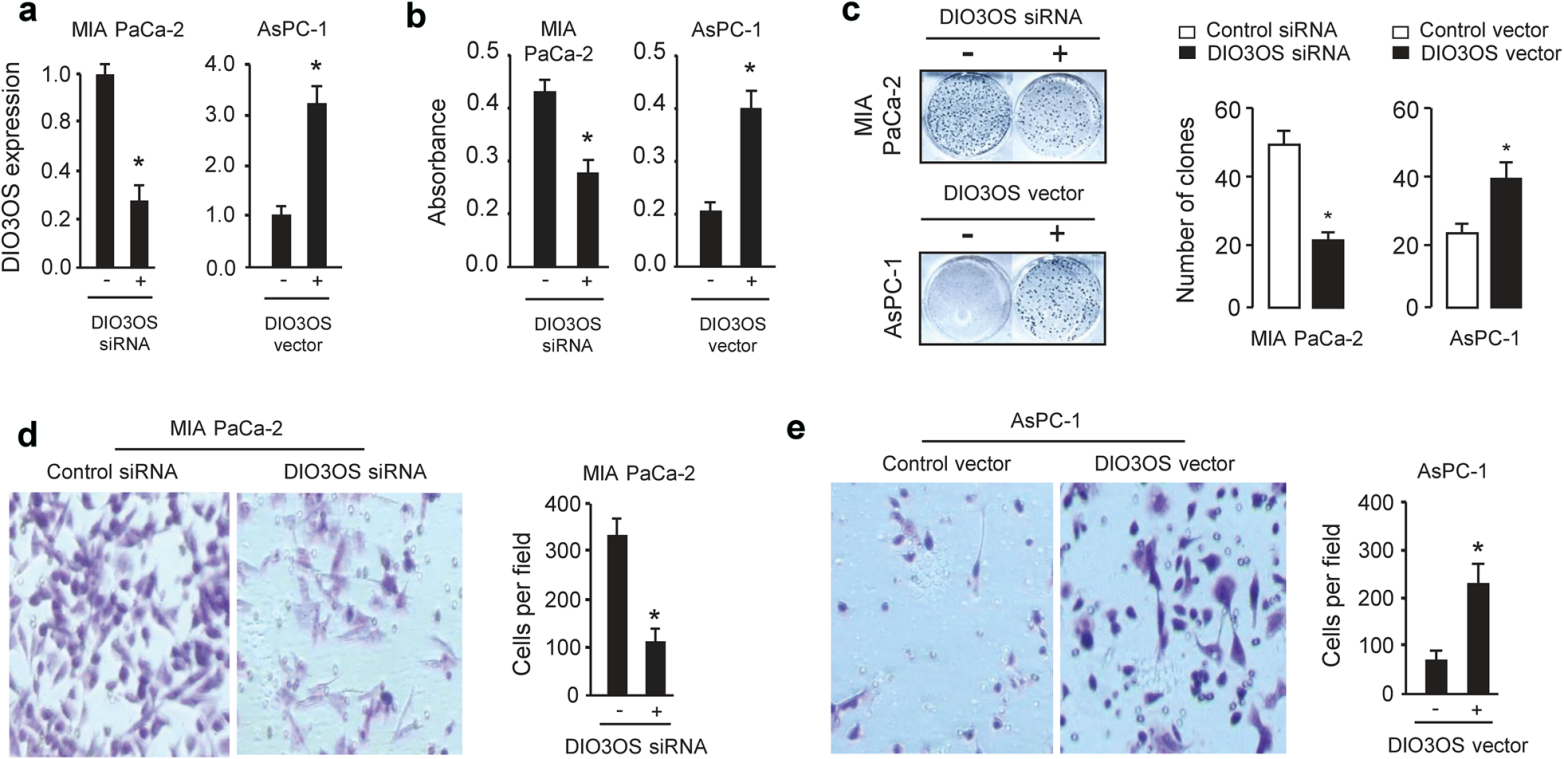
DIO3OS stimulates proliferation and invasion of PC cells. **a** qRT-PCR analysis of DIO3OS expression in MIA PaCa-2 cells after DIO3OS knockdown and in AsPC-1 cells after overexpression of DIO3OS. **b** PC cells were transfected with DIO3OS siRNA or control siRNA, DIO3OS vector or control vector, and cell proliferation was measured using a CCK-8 assay. **c** PC cells were transfected with DIO3OS siRNA or control siRNA, DIO3OS vector or control vector, and cell proliferation was measured using a clone formation assay. **d** Invasion of MIA PaCa-2 cells was examined after DIO3OS knockdown. **e** Invasion of AsPC-1 cells was examined after overexpression of DIO3OS. *P < 0.05

The effect of DIO3OS on the ability of PC cells to proliferate and invade after transfection with DIO3OS siRNA or control siRNA was then characterized using CCK-8 assay, clone formation assays and Matrigel invasion assays. CCK-8 assays, clone formation assays and Matrigel invasion assays revealed that the knockdown of DIO3OS significantly decreased cell proliferation and invasion in MIA PaCa-2 cells (Fig. 2a–d). To further explore the oncogenic roles of DIO3OS in PC, we overexpressed DIO3OS in AsPC-1 cells by transfecting AsPC-1 cells with the DIO3OS expression vector (Fig. 2a). CCK-8 assays, clone formation assays and invasion assays showed that the overexpression of DIO3OS can promote proliferation and invasion in AsPC-1 cells (Fig. 2b, c and e), suggesting a critical role for DIO3OS in promoting growth and invasiveness of PC cells.

### DIO3OS sponges miR-122 to promote cell proliferation and invasion in PC

**Fig. 3:**
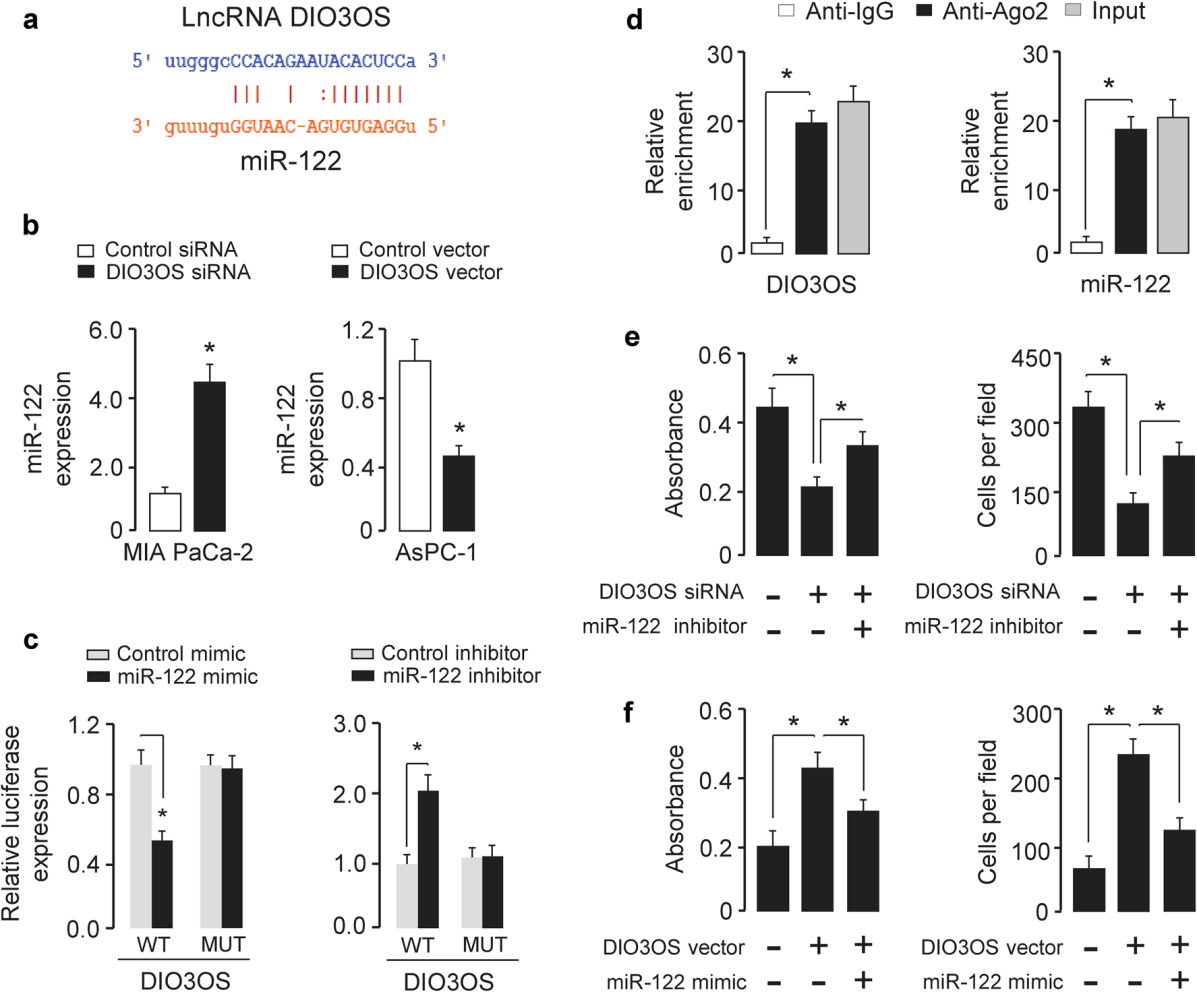
DIO3OS acts as a molecular sponge for miR-122. **a** The predicted binding site of miR-122 to the DIO3OS sequence. **b** Relative expression of miR-122 in MIA PaCa-2 cells after DIO3OS knockdown and in AsPC-1 cells after overexpression of DIO3OS. **c** Luciferase reporter gene assays were used to confirm the direct binding between miR-122 and DIO3OS. **d** Lysates of MIA PaCa-2 cells underwent RIP assay with the Ago2 antibody. Expression of DIO3OS and miR-122 was detected using qRT-PCR analysis. Results are shown as fold enrichment of Ago2 relative to IgG immunoprecipitates. **e** DIO3OS siRNA or control siRNA was transfected into MIA PaCa-2 cells, together with (or without) miR-122 inhibitor. The cells were assayed for cell proliferation (left) and invasion (right). **f** DIO3OS vector or control vector was transfected into AsPC-1 cells, together with (or without) miR-122 mimic. The cells were assayed for cell proliferation (left) and invasion (right). *P < 0.05

Several studies have identified that lncRNAs might act as a sponge for miRNAs [12]. To examine whether DIO3OS regulates the expression of miRNAs in such a manner, interactions between DIO3OS and miRNAs were predicted using starBase v2.0 [13]. Interestingly, this bioinformatics analysis of miRNAs target sequences on DIO3OS revealed that miR-122 was complementary to DIO3OS sequence (Fig. 3a). To test whether DIO3OS can regulate miR-122 expression, we investigated the effect of DIO3OS knockdown or overexpression on miR-122 expression in PC cells. Our qRT-PCR analysis suggested that miR-122 expression was upregulated in MIA PaCa-2 cells when DIO3OS was knockdown (Fig. 3b). However, the overexpression of DIO3OS downregulated the expression of miR-122 in AsPC-1 cells (Fig. 3b). Then, miR-122 expression levels in two PC cell lines and the normal renal cell line HPDE6-C7 were investigated. Our qRT-PCR results showed an inverse correlation between DIO3OS and miR-122 expression in PC cell lines (Fig. 1c, e).

To confirm whether DIO3OS can interact with miR-122, the fragment of DIO3OS was inserted downstream of the luciferase gene and a dual-luciferase reporter assay was performed to explore the relationship between DIO3OS and miR-122. When miR-122 was overexpressed, the luciferase activity of wild-type DIO3OS was significantly reduced (Fig. 3c). To further determine whether there is a direct interaction between miR-122 and DIO3OS putative binding site, we mutated the miR-122 binding site by site-directed mutagenesis, and found that mutations in the DIO3OS abrogated the repressive effect of miR-122 (Fig. 3c). As expected, the transfection with miR-122 inhibitor significantly increased in the luciferase activity of wild-type DIO3OS in AsPC-1 cells, while the luciferase activity of mutant DIO3OS was unaffected (Fig. 3c).

To investigate whether DIO3OS and miR-122 are part of the RNA-induced silencing (RISC) complex, RIP experiment was conducted on MIA PaCa-2 cells lysates using an antibody against Ago2, a key component of the RISC complex. When DIO3OS and miR-122 levels were quantified in the immunoprecipitates using qRT-PCR analysis, they were found to be enriched in Ago2 immunoprecipitates compared to control IgG immunoprecipitates (Fig. 3d).

To examine if miR-122 was capable of counteracting the DIO3OS-mediated promotion of cell proliferation and invasion, MIA PaCa-2 cells were transfected with DIO3OS siRNA in combination with miR-122 inhibitor, and AsPC-1 cells were transfected with the DIO3OS vector in combination with miR-122 mimic. The inhibition of miR-455 rescued the effects of DIO3OS silencing on MIA PaCa-2 cell proliferation and invasion (Fig. 3e). Moreover, the overexpression of miR-455 overcame the effects of DIO3OS overexpression in AsPC-1 cells (Fig. 3f). Taken together, these results provide evidence that miR-122 was involved in DIO3OS-mediated regulation of PC cell proliferation and invasion.

### miR-122 directly targets ALDOA in PC cells

**Fig. 4:**
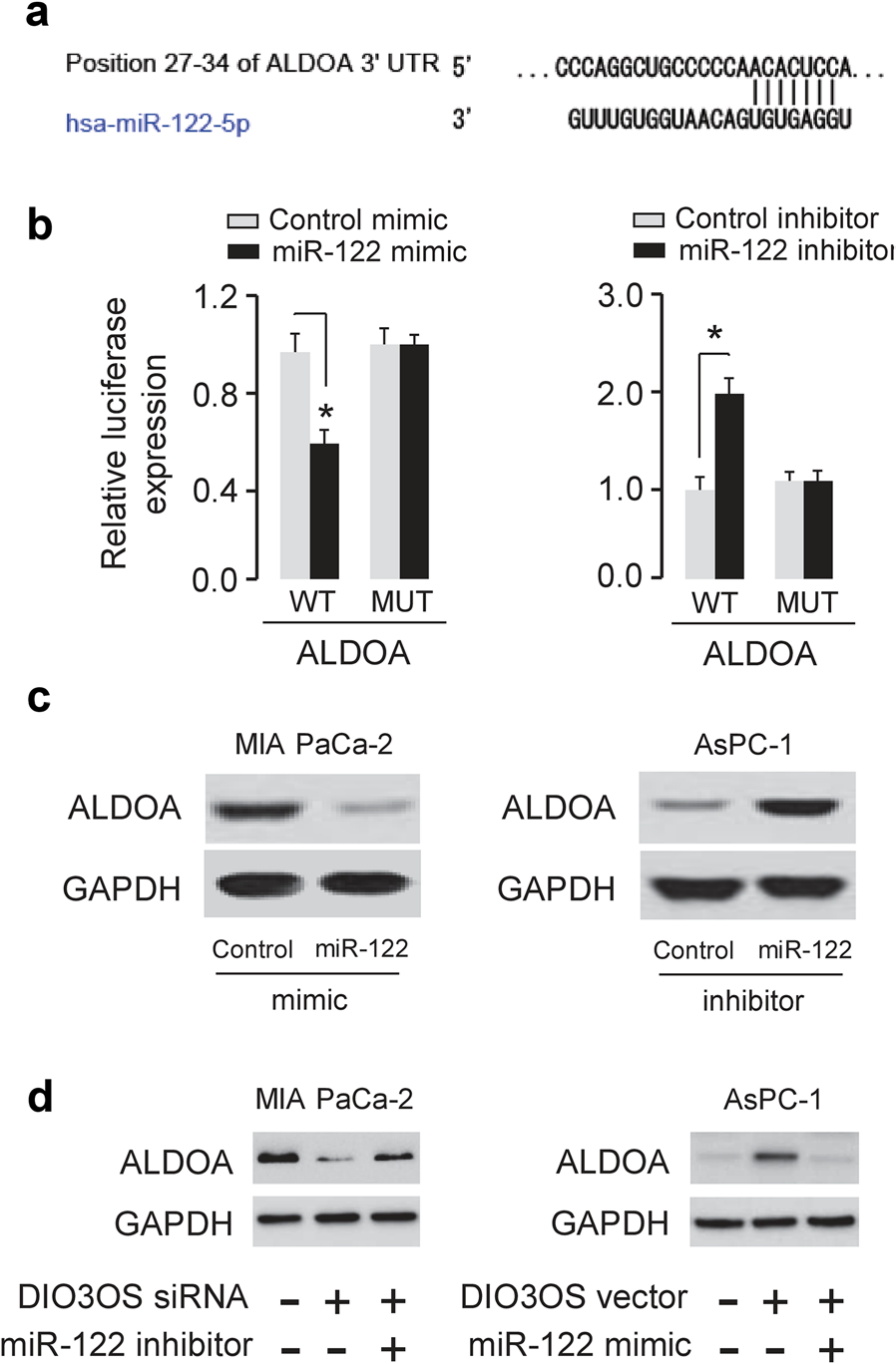
DIO3OS increases ALDOA expression via inhibiting miR-122 expression. **a** Predicted miR-122 binding site in the 3′-UTR of ALDOA. **b** MIA PaCa-2 cells (left) were transfected with luciferase reporter vectors containing wild-type (WT) ALDOA or mutant (MUT) ALDOA, together with (or without) miR-122 mimic, and AsPC-1 cells (right) were transfected with luciferase reporter vectors containing wild-type ALDOA or mutant ALDOA, together with (or without) miR-122 inhibitor. Luciferase reporter gene assays were used to evaluate the interaction between miR-122 and ALDOA. **c** The protein expression of ALDOA in MIA PaCa-2 cells transfected with miR-122 mimic or control mimic, and in AsPC-1 cells transfected with miR-122 inhibitor or control inhibitor. **d** The protein expression of ALDOA in MIA PaCa-2 cells transfected with DIO3OS siRNA or control siRNA, together with (or without) miR-122 inhibitor, and in AsPC-1 cells transfected with DIO3OS vector or control vector, together with (or without) miR-122 mimic. The cropped blots are used in the figure. *P < 0.05

We explored the genes that were potentially regulated by miR-122. Our bioinformatic-based target prediction analysis using TargetScan (http://www.targetscan.org) indicated that ALDOA was predicted to contain the binding sequence of miR-122 (Fig. 4a). Consistently, the protein expression of ALDOA was elevated in AsPC-1 and MIA PaCa-2 cells compared to the normal pancreatic cell line HPDE6-C7 (Fig. 1f), supporting a negative correlation between miR-122 and ALDOA expression.

As shown in Fig. 4b, luciferase assays showed the ectopic expression of miR-122 could significantly decrease the luciferase activity of the wild-type ALDOA 3′-UTR, but not that of the mutant ALDOA 3′-UTR. In contrast, the knockdown of miR-122 significantly enhanced the luciferase activity of the wild-type ALDOA 3′-UTR, but did not affect the luciferase activity of the mutant ALDOA 3′-UTR (Fig. 4b). Western blot analysis demonstrated that overexpression of miR-122 could reduce the levels of ALDOA. However, the inhibition of miR-122 increased the protein expression of ALDOA in PC cells (Fig. 4c). These data suggested that miR-122 directly binds to ALDOA 3′-UTR and represses ALDOA expression level in PC cells.

Then, we asked whether DIO3OS might regulate the level of ALDOA through inhibiting miR-122 expression. Western blot analysis revealed that decreased expression of ALDOA by DIO3OS knockdown could be partially rescued by miR-122 inhibitor, and the induction of ALDOA expression caused by DIO3OS overexpression could be largely reduced by miR-122 mimic in AsPC-1 cells (Fig. 4d), suggesting that DIO3OS enhances ALDOA expression through negative modulation of miR-122.

### ALDOA mediates the effects of DIO3OS and miR-122 in PC cells

**Fig. 5:**
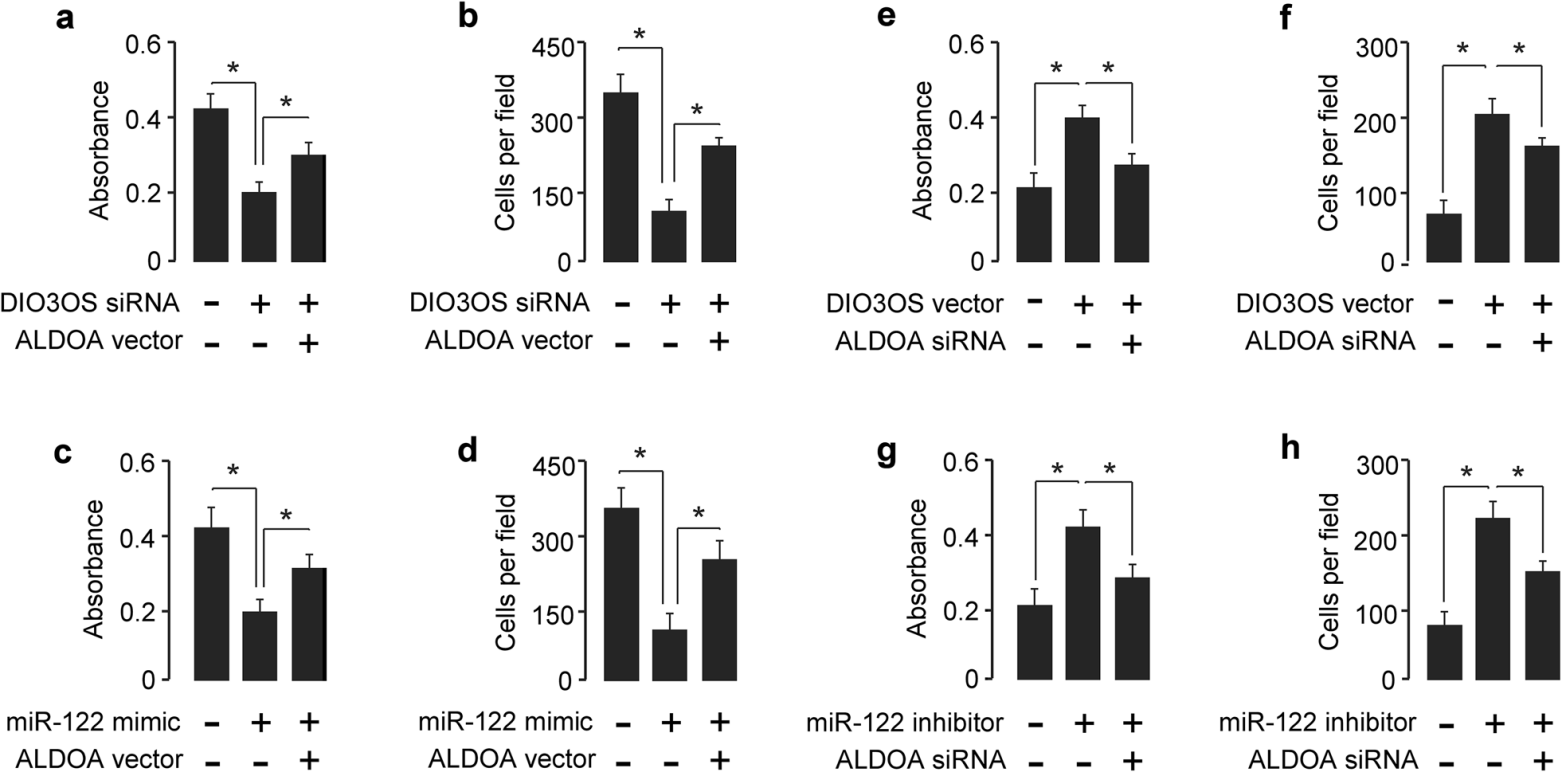
ALDOA restoration reverses the suppression of cell proliferation and invasion mediated by DIO3OS knockdown or miR-122 overexpression. **a**, **b** Cell proliferation (**a**) and invasion (**b**) was assessed in MIA PaCa-2 cells transfected with DIO3OS siRNA or control siRNA, together with (or without) ALDOA vector. **c**, **d** Cell proliferation (**c**) and invasion (**d**) was assessed in MIA PaCa-2 cells transfected with miR-122 mimic or control mimic, together with (or without) ALDOA vector. **e**, **f** AsPC-1 cells were transfected with DIO3OS vector or control vector, together with (or without) ALDOA siRNA, and cell proliferation (**e**) and invasion (**f**) were measured. **g**, **h** AsPC-1 cells were transfected with miR-122 inhibitor or control inhibitor, together with (or without) ALDOA siRNA, and cell proliferation (**g**) and invasion (**h**) were measured. *P < 0.05

We investigated whether the functions of DIO3OS and miR-122 in MIA PaCa-2 cells were dependent on the expression of ALDOA. CCK-8 and invasion assays showed that the suppression of cell proliferation and invasion by DIO3OS knockdown or miR-122 overexpression could be restored by the ectopic expression of ALDOA (Fig. 5a–d). On the other hand, the increased proliferation and invasion of AsPC-1 cells by DIO3OS overexpression or miR-122 inhibition was suppressed by the knockdown of ALDOA (Fig. 5e–h).

Importantly, to further confirm the existence of the DIO3OS/miR-122/ALDOA axis in PC tissues, we detected the levels of miR-122 and ALDOA in PC samples and non-tumor samples obtained from the TCGA database using the MethHC database and UALCAN web server, respectively. We found that the level of miR-122 was downregulated, while the expression of ALDOA was upregulated in PC tissues compared with normal samples (Fig. 1g, h), indicating a negative correlation between DIO3OS and miR-122 expression (Fig. 1b, g), and a negative association between miR-122 and ALDOA expression in PC samples (Fig. 1g, h). These data suggested that ALDOA mediates the effects of DIO3OS and miR-122 in PC cells.

### DIO3OS facilitates PC growth in vivo

**Fig. 6:**
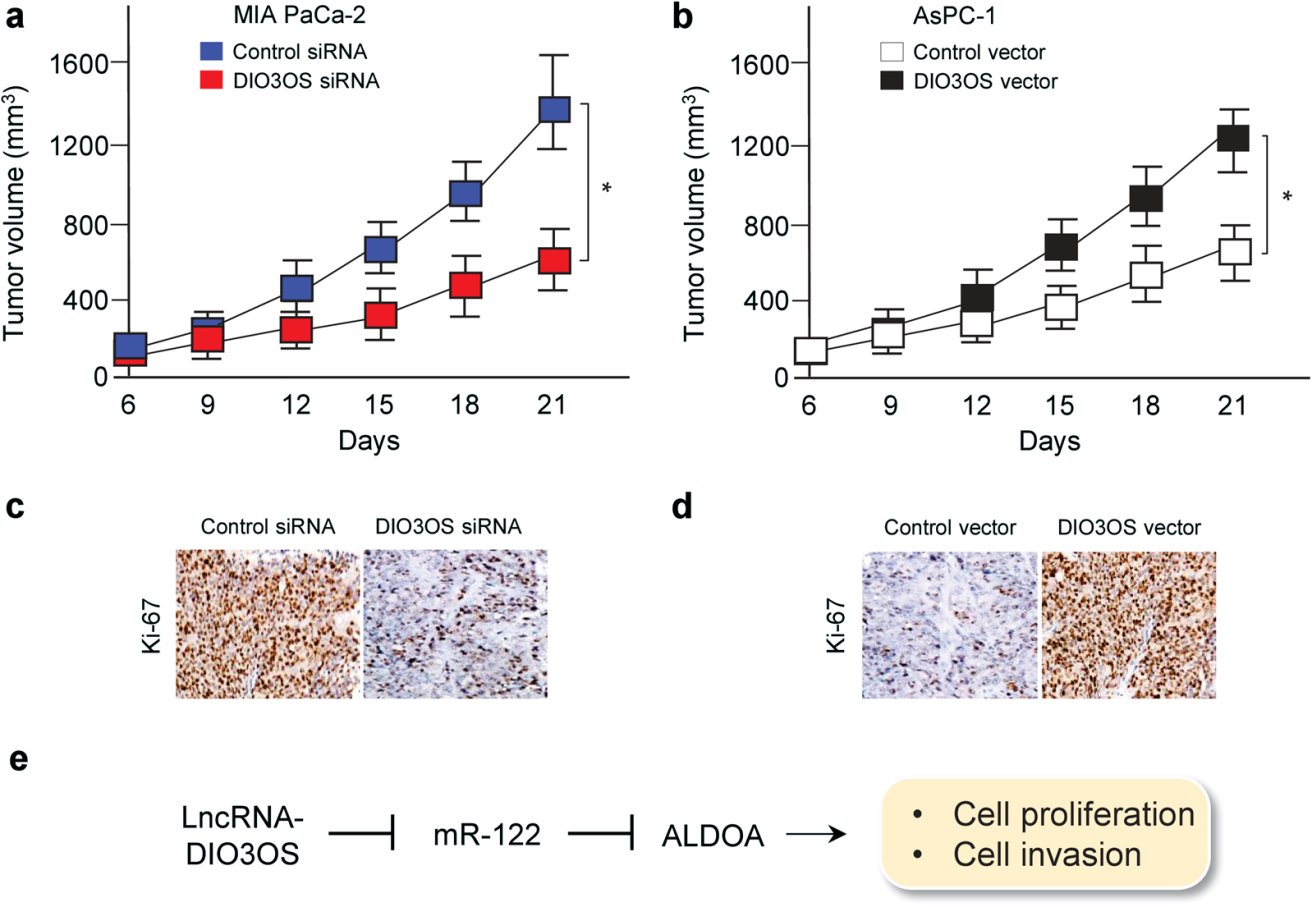
DIO3OS promotes PC growth in vivo. **a**, **b** Nude mice were subcutaneously injected with MIA PaCa-2 cells transfected with DIO3OS siRNA or control siRNA (**a**), or AsPC-1 cells transfected with DIO3OS vector or control vector (**b**). After implantation for 6 days, tumor volume was measured every 3 days (n = 4); **c**, **d** Immunohistochemistry analysis of Ki-67 protein levels in xenograft tumor tissues from the mice described in (**a**) and (**b**). **e** The proposed mechanisms by which DIO3OS promotes PC cell growth and invasion via regulating the miR-122/ALDOA axis. *P < 0.05

To further investigate whether DIO3OS promotes PC growth in vivo, we knocked down the expression of DIO3OS in MIA PaCa-2 cells or overexpressed DIO3OS in AsPC-1 cells, and injected these cells into the flanks of nude mice to establish subcutaneous PC xenografts. As shown in Fig. 6a, b, the depletion of DIO3OS could suppress the tumor growth, and the overexpression of DIO3OS significantly increased tumor growth. Furthermore, the knockdown of DIO3OS downregulated the expression of Ki-67, a cell proliferation marker (Fig. 6c), and the overexpression of DIO3OS increased the levels of Ki-67 in vivo (Fig. 6d). Collectively, these results showed that DIO3OS promotes PC cell growth in vivo. Taken together, our results support the notion that DIO3OS promotes PC cell growth and invasion via regulating the miR-122/ALDOA axis (Fig. 6e).

## Discussion

Mounting pieces of evidence showed that lncRNAs play important roles in tumor progression and might be used as a diagnostic and therapeutic target. DIO3OS is a newly identified lncRNA [4]. In the current research, we found for the first time that DIO3OS markedly promoted PC cell proliferation and invasion in vitro and facilitated PC growth in vivo. These data suggested that DIO3OS played a critical role in the biological function of PC development.

LncRNAs might exert their functions as ceRNAs [3]. Thus, to explore the mechanism by which DIO3OS functions in PC, we searched for candidate miRNAs and confirmed that DIO3OS could compete with miR-122 and other miRNAs to tumor growth and invasion. The potential miRNAs that might interact with DIO3OS should be investigated in future works.

The expression of lncRNAs can be regulated both at the transcriptional and post-transcriptional levels [14]. In addition, miRNAs can regulate the expression of lncRNAs for degradation by targeting them for degradation via the RNA-induced silencing complex [15]. However, the detailed mechanisms that can explain DIO3OS induction in PC cells are unclear. In this study, we reported that miR-122 directly bond to DIO3OS and inhibited its expression in PC cells. Therefore, our study supported a DIO3OS-miRNA feedback loop, which enhanced DIO3OS expression and reduced miR-122 expression, leading to the acceleration of PC progression.

Recent studies demonstrated that miR-122 inhibited cell proliferation, migration, and invasion in several tumors [16–19]. Although miR-122 was shown to be downregulated in PC [20], its role in PC cells remains unclear. Our data support that miR-122 served as a novel tumor suppressor in PC by controlling the expression of DIO3OS. The mechanisms by which miR-122 suppresses PC progression, remains largely elusive.

Abnormal metabolism has been widely regarded as a general hallmark of human cancer, and cancer cells frequently exhibit increased glycolysis and depend largely on this metabolic pathway for the generation of energy, even in the presence of oxygen [21]. ALDOA, a glycolytic enzyme that catalyzes the reversible conversion of fructose-1, 6-bisphosphate to glyceraldehyde-3-phosphate and dihydroxyacetone phosphate, was shown to be aberrantly expressed in multiple cancer types [22]. However, accumulated studies have proven that ALDOA also promotes cancer growth and metastasis through its non-enzymatic functions [22]. In PC cells, ALDOA has been shown to promote the proliferation and metastasis of PC cells [23]. Our current study confirmed that ALDOA played critical roles in PC cells, in particular, by controlling cell proliferation and invasion. Importantly, we found that ALDOA was a direct target of miR-122. This finding was consistent with the previous notion that miR-122 can inhibit glycolysis and spheroid formation at least by targeting PDK4 in hepatocellular cancer [24]. It would be interesting to further determine the possible roles of the DIO3OS/miR-122/ALDOA axis in regulating glycolysis and cancer progression in PC.

## Conclusions

In conclusion, our study revealed that DIO3OS is upregulated in PC and can promote the proliferation and invasion of PC cells via the miR-122/ALDOA axis. Therefore, the DIO3OS/miR-122/ALDOA axis has potential as an effective therapeutic target against PC.

## Data Availability

The data are included within the manuscript.

## Abbreviations

PC: pancreatic cancer
lncRNA: long non-coding RNAs
miRNAs: microRNAs
ceRNA: competing endogenous RNA
